# Effect of Selection for Litter Size Variability on Growth, Carcass and Meat Quality in Rabbits

**DOI:** 10.3390/vetsci12020160

**Published:** 2025-02-12

**Authors:** Ireneusz Zawiślak, Maria-Jose Argente, Katarzyna Leicht, Iván Agea, Maria de la Luz García, Rafik Belabbas, Małgorzata Korzeniowska

**Affiliations:** 1 Department of Functional Food Product Development, Faculty of Biotechnology and Food Science, Wroclaw University of Environmental and Life Sciences, 50-375 Wroclaw, Poland; ireneusz.zawislak@upwr.edu.pl (I.Z.); katarzyna.leicht@upwr.edu.pl (K.L.); malgorzata.korzeniowska@upwr.edu.pl (M.K.); 2 Instituto de Investigación e Innovación Agroalimentaria y Agroambiental (CIAGRO-UMH), Universidad Miguel Hernández de Elche, Ctra. de Beniel km 3.2, 03312 Orihuela, Spain; iagea@umh.es (I.A.); mariluz.garcia@umh.es (M.d.l.L.G.); 3 Laboratory of Research “Health and Animal Productions”, Higher National Veterinary School, Road Issad 26 Abes, Oued Smar, Algiers 16200, Algeria; r.belabbas@ensv.dz

**Keywords:** pH, protein content, fatty acids, energy value, thiobarbituric acid, rabbit

## Abstract

This study aimed to analyze the effect of reducing litter size variability on growth rate, carcass traits, and meat quality in rabbits. Two divergent lines selected by litter size variability were compared: a line selected to reduce litter size variability (HO) and one selected to increase litter size variability (HE). All animals were fed the same commercial diet and kept under identical rearing conditions. The HO line had lower live weight at weaning compared to the HE line (*p* ≤ 0.05), but the growth rate was higher in the HO line than in the HE line (*p* ≤ 0.05). Carcass weight and composition were similar between the lines. No differences in meat pH at 45 min and 24 h post-mortem were found between lines. However, the HO line had a higher lightness (L*) value at 45 min post-mortem than the HE line (*p* ≤ 0.05). The HO line showed higher Ca concentrations than the HE line (*p* ≤ 0.05). The HO line also had higher protein content and lower saturated fatty acid content than the HE line (*p* ≤ 0.05). No differences in fat oxidation were observed among the lines. In conclusion, the selection to reduce litter size variability did not negatively impact growth rate and had a favorable effect on protein content, as well as a reduction in saturated fatty acid content in carcass.

## Simple Summary

This study analyzed the effect of selection to reduce litter size variability on growth rate, carcass composition, and meat quality in rabbits. Reducing litter size variability improved meat lightness, as well as protein and calcium content, decreased saturated fatty acid levels, and had no adverse effects on carcass composition and meat pH.

## 1. Introduction

Rabbit meat is a highly nutritious and digestible food source, rich in high-quality proteins containing all essential amino acids, making it ideal for supporting muscle growth and repair [1,2]. Compared to other types of meat, rabbit meat is lower in fat and cholesterol and has a favourable profile of polyunsaturated fatty acids (PUFAs), including omega-3 and omega-6, which support cardiovascular health and reduce inflammation [3,4]. Its low-calorie content and high nutrient density make it suitable for weight management [5], while its abundance of vitamins (B12, E) and minerals (iron, zinc, phosphorus, selenium) is essential for energy metabolism, immune function, and oxygen transport [6]. These properties, along with their suitability for sensitive digestive systems, position rabbit meat as a healthy alternative to red meats in the human diet [7].

Despite the health-promoting properties of rabbit meat, global per capita consumption is only 0.19 kg/year, a small amount compared to beef (8.98 kg/year) or sheep and goat (1.96 kg/year) [8]. The average per capita consumption of rabbit meat varies by country, ranging from 6.81 kg/person/year in the Democratic People’s Republic of Korea to 0.44 kg/person/year in Germany, with the European Union’s average at 0.51 kg/year [9]. Therefore, investigating the technological properties of rabbit meat is important, particularly for its incorporation into the human diet, since this can inform the creation of value-added products such as roasted, smoked, canned, frozen, cured, dried, sauce, or pickled goods and burger and sausage-type products containing rabbit meat [10].

Modifying the diet of rabbits can produce meat with functional food properties, characterized by high levels of polyunsaturated fatty acids, eicosapentaenoic acid, docosahexaenoic acid, conjugated linoleic acid, vitamin E, selenium, and other bioactive compounds, such as essential amino acids and a lower n-6/n-3 ratio [1,2]. Additionally, the genetic origin of rabbits can influence meat quality [11,12]. In the rabbit meat industry, commercial maternal lines are selected for reproductive efficiency, while commercial paternal lines are selected for growth rate or body weight at a stage close to market age [13]. Selection for increasing litter size may increase intrauterine competition among fetuses [14], which can potentially affect postnatal development [13].

Our team at Universidad Miguel Hernández de Elche (Orihuela, Alicante, Spain) has conducted a divergent selection experiment over sixteen generations to increase (the HO line) and decrease (the HE line) litter size homogeneity. The litter size variability was estimated within the females as the phenotypic variance of the number of kits per parity after correcting for the effects of year/season and parity-lactation status [15]. Increased homogeneity in litter size is directly associated with greater reproductive efficiency; in this regard, the average litter size was 8.3 kits at birth in the HO line and 7.1 kits at birth in the HE line [16]. Furthermore, higher homogeneity in litter size positively affects milk supply to each kit, thereby improving growth and productive performance [17]. However, the potential impact of increasing litter size homogeneity on growth, carcass quality, and meat quality remains unclear.

The objective of this study was to analyze the effect of selection on reducing litter size variability on growth rate, as well as on carcass and meat quality in rabbits. For this purpose, growth rate, carcass weight, pH, color, mineral content, protein, fat and energy, antioxidant properties, and fatty acid profile were compared between the HO and HE lines. In divergent selection experiments, each line acts as a control for the other. As suggested by Pascual et al. [18], and due to both the HO and HE lines exhibiting similar weights upon reaching adulthood [19], comparison at the same age can be used as a good approximation.

## 2. Materials and Methods

All experimental procedures were approved by the Miguel Hernández University of Elche Research Ethics Committee, according to Council Directives 98/58/EC and 2010/63/EU (reference 230518145656).

### 2.1. Animals and Management

The experiment was conducted at the Universidad Miguel Hernández de Elche (Orihuela, Alicante, Spain). Carcass composition and meat quality evaluations were performed on 60 animals (30 from each of the HO and HE lines) at 63 days of age, which corresponds to the usual slaughter age in the Spanish market.

After weaning at 30 days of age, animals were identified and weighed. Young rabbits were housed in collective cages (37.5 cm × 33 cm × 90 cm), with six rabbits per cage, from weaning (30 days) until 9 weeks of age (63 days). Animals were kept under the same conditions, with a constant photoperiod of 16:8 h and controlled ventilation. Water and feed were available ad libitum. Animals were fed a pelleted commercial diet formulated by the NANTA S.A. (Las Palas, Murcia, Spain) with 212 g crude fiber, 163 g crude protein, and 25 g ether extract per kg of feed. At the end of the experiment, the rabbits were weighed and slaughtered at 63 days of age at the slaughterhouse of the Cunicultura de la Manchuela Ltd. The rabbits were fasted overnight and slaughtered the following morning by electric stunning and bleeding from a jugular cut. The growth rate was estimated as weight gain from 30 to 63 days divided by 33 days.

### 2.2. Slaughtering and Dissection Procedure

After slaughter, the carcasses, which included the head, heart, lungs, liver, kidneys, and fat deposits, were hung for 30 min in a ventilated room and then chilled at 3–4 °C until 24 h post-mortem. The reference carcass weight, consisting of only the meat, fat, and bone, was then determined by removing the head, liver, lungs, thymus, trachea, esophagus, heart, and kidneys. The carcasses were dissected following the guidelines of the World Rabbit Scientific Association [20]. The whole forelegs, loin, and hind legs were stored at −80 °C for further analysis. The following traits were recorded: the chilled carcass weight at 45 min and 24 h post-mortem; the reference carcass weight (chilled carcass without the head and organs); dressing percentage ([chilled carcass weight at 24 h post-mortem/live weight at slaughter] × 100%); and the percentages ([weight/chilled carcass weight at 24 h post-mortem] × 100%) of interscapular fat, perirenal fat, inguinal fat, liver, kidneys, forelegs, thoracic cage, loin, and hind part.

### 2.3. Meat Quality

The meat color in the CIELAB space (L*, color lightness; a*, redness; b*, yellowness) of the carcasses was measured in the epimysium of the loin at the 4th lumbar vertebra of the left side and in the *Biceps femoris* of the hind legs at 45 min and 24 h post-mortem using a CR300 Minolta Chromameter with a DP-301 Data Processor (Konica Minolta Sensing Americas Inc., Ramsey, NJ, USA). The pH was measured at 45 min and 24 h post-mortem in the loin at the 4th lumbar vertebra of the left side and hind legs with a Crison MicropH GLP 21 (Crison instruments, Barcelona, Spain) using a combined electrode penetrating to 3 mm. Each measurement of color and pH was repeated two times.

### 2.4. Minerals

To determine the trace elements, a closed quartz vessel and microwave oven digestion procedure were applied [21]. Specifically, 1 g of meat from the loin and the *Biceps femoris* of the hind legs, which are considered to be most representative of rabbit meat quality [20], was accurately weighed into quartz digestion vessels. Two milliliters of hydrogen peroxide (H_2_O_2_ 30% pro analysis, Merck, Darmstadt, Germany) and 8 mL of 65% nitric acid (HNO_3_ Suprapur, Merck, Darmstadt, Germany) were added to each tube, which was then closed. After cooling, the digests were transferred into 50 mL volumetric flasks and filled to the mark using type 1 water (obtained from a Milli-Q water purification system, Millipore, Molsheim, France). Next, 3 mL of the solution was filtered using 0.45 μm nylon filters, and 2 mL of the filtrate was removed and incorporated into a test tube with 8 mL of type I water, resulting in a 1:5 dilution of the sample. The macro- and micronutrients (macro: Ca, Na, K, and Mg; micro: Fe, Cu, Mn, and Zn) present in the diluted sample were then quantitatively determined using *Inductively Coupled Plasma Mass Spectrometry* (CPMS-2030 Shimadzu, Tokyo, Japan). Calibration lines for the elements were established prior to measurement, and each measurement was repeated two times.

### 2.5. Protein Analysis

The protein content was determined in 1 g of the homogenized dried meat of the forelegs, loin, and hind legs using the Kjeldahl method according to PN-75/A-04018 with modification according to PN-75-A-04018/Az3:2002 [22]. The results were converted to grams per 100 g of fresh sample weight, and each of the subsequent measurements was repeated three times.

### 2.6. Fat Analysis

The fat content was determined 1 g of the homogenized dried meat of the forelegs, loin, and hind legs using the Soxhlet extraction method and according to PN-ISO 1444:2000 [23]. The results were converted to grams per 100 g of fresh sample weight. Each of the subsequent measurements was repeated three times.

### 2.7. Fatty Acid Profile Analysis

The fat present in the homogenized meat of the forelegs, loin, and hind legs was extracted using Folch’s method, and methyl esters of fatty acids were obtained according to described procedures [24] using a 2 M KOH solution in methanol. A gas chromatograph coupled with a mass spectrometer (GC6890-MS5893, Agilent Technologies Inc., Santa Clara, CA, USA) with a quadrupole mass detector was used to analyze the fatty acid profile. The analyzed mixture was separated using an HP-88 capillary column (0.25 mm × 100 m) packed with a 0.2 μm-grain size cyanopropyl-aryl-polysiloxane bed (88:12). Helium was used as the mobile phase, injected in a split mode (split 4:1) at a flow rate of 1 mL/min. The temperature program was run for a total of 50.33 min as follows: an initial temperature of 60 °C was maintained for 2 min; it was increased by 3 °C/min to 220 °C and held for 15 min; it was followed by heating at a rate of 5 °C/min to 250 °C, which was maintained for 8 min. The results are presented as the percentage of the total fatty acid content in the analyzed meat sample. Each of the subsequent measurements was repeated three times.

### 2.8. Degree of Fat Oxidation Analysis

The degree of fat oxidation in the homogenized meat of the forelegs, loin, and hind legs was determined using thiobarbituric acid (TBARS) method, described by Piette and Raymond [25]. Specifically, 1 g of the sample was weighed, and 10 mL of 10% TBA was added. The mixture was homogenized using an IKA T 18 digisl Ultra-Turrax Homogenizer (IKA, Staufen, Germany) and then centrifuged at 4000× *g* for 10 min. From the resulting supernatant, 2 mL was washed and transferred to a Falcon test tube. Then, 2 mL of 0.02 M thiobarbutyric acid was added. The samples were incubated for 40 min at 95–100 °C in a Julabo EcoTemp TW 12 water bath (Julabo, Seelbach, Germany). After 20 min of cooling under tap water, a UV-1800 spectrophotometer (Shimadzu, Kyoto, Japan) was used to measure the absorbance at λ = 530 nm, with distilled water as the blank. The results were calculated using the concentration of malonic dialdehyde (1,1,3,3-tetra-methoxypropane; MDA) and a standard calibration curve, and they were expressed as mg MDA per kg of meat. Each of the subsequent measurements was repeated three times.

### 2.9. Dry Weight Analysis

The dry weight of the homogenized meat from the forelegs, loin, and hind legs was measured using approximately 5 g of fresh tissue, with measurements recorded to the fourth decimal place; the vessels were weighed together with the raw meat. Then, the samples were dried in a top-loading Beschickung Modell 100-800 electric dryer (Memmert, Dresden, Germany) at 103 °C for 24 h. After drying, the samples were cooled to room temperature in a desiccator. Then, the vessels were weighed again with the dried meat. The analysis was performed according to PN-73/A-82110 [26]. The dry weight of the meat was calculated using the following formula:

dry weight (%) = (A − C)/(B − C) × 100%

where A is the weight of the vessel and dried meat, B is the weight of the vessel and raw meat, and C is the weight of the empty vessel. Each of the subsequent measurements was repeated three times

### 2.10. Ash Analysis

The ash content of the previously homogenized meat samples from the forelegs, hind legs, and loin was determined according to PN-ISO 936:2000 [27]. The results were converted to grams per 100 g of fresh sample weight. Each of the subsequent measurements was repeated three times.

### 2.11. Energy Value Analysis

The previously homogenized meat from the forelegs, loin, and hind legs was dried in a top-loading Beschickung Modell 100-800 electric laboratory dryer at 103 °C for 24 h. The dried samples were rehomogenized, and ~1 g of each sample was weighed. The energy value of the weighed samples was assessed using a KL-14 computer microprocessor calorimeter (EDCO, Frederik, MD, USA). The results were converted to kcal per 100 g of fresh sample weight. Each of the subsequent measurements was repeated three times.

### 2.12. Statistical Analysis

The growth and carcass data were analyzed using a model that included the line (with two levels: the HO and HE) as fixed effects. The model included a random animal effect and measuring part effect (forelegs, loin, and hind legs) for the remaining traits. The PROC GLM of SAS [28] was used to analyze the growth and carcass traits, and the remaining traits were analyzed with PROC MIXED of SAS [28]. The MIXED model allowed for a nested animal effect with repeated measures. The differences between the groups were determined using Tukey’s multiple comparison test. The results are reported as means, and the significance was set at *p* < 0.05.

## 3. Results

### 3.1. Growth Performance and Carcass Traits

Table 1 shows the live weight, growth rate from weaning to slaughter, carcass characteristics, as well as the proportions of meat parts and fat in the two divergent lines selected for litter size variability. The kits from the HO line were 43% lighter at weaning (30 days) than those from the HE line (*p* ≤ 0.05) when the groups were formed. At slaughter (63 days of age), the difference was reduced to 10% due to a higher growth rate in the kits of the HO line (*p* ≤ 0.05). The difference in slaughter weight disappeared when weight at weaning was included in the model as a covariate, indicating that the difference between lines for slaughter weight was due to the lower weight at weaning in the kits from the HO line. Additionally, the HO line showed similar weights for the chilled carcass at 45 min and 24 h post-mortem, as well as for the reference carcass, compared to the HE line. No significant differences were observed between the lines when the weights of different body parts (liver, kidneys, forelegs, thoracic cage, loin, hind legs, and fat) were analyzed as a proportion of the total carcass weight (Table 1).

### 3.2. Meat Traits

Table 2 shows the pH and L*, a*, and b* values at 45 min and 24 h post-mortem in the meat of the two divergent lines selected by litter size variability, as well as in the hind legs and loin. The pH values at 45 min and 24 h post-mortem were similar between the lines. The hind legs had a lower pH value at 45 min post-mortem and a higher value at 24 h post-mortem compared to the loin (6.45 vs. 6.83, respectively, at 45 min post-mortem; 5.85 vs. 5.66 at 24 h post-mortem, *p* ≤ 0.05). The HO line showed higher meat lightness (L*) at 45 min post-mortem than the HE line (*p* ≤ 0.05). No differences were observed in a* (redness) and b* (yellowness) between the lines. However, the meat of the hind legs exhibited higher a* values at 45 min and 24 h post-mortem (*p* ≤ 0.05), indicating a darker red color in comparison with the meat from the loin. In relation to macro- and microminerals concentration in meat, no differences were found in concentrations of K, Na, Mg, Zn, Fe, Cu, and Mn among lines (see Table 3). However, Ca concentration was higher in the HO line than in the HE line (17.50 mg/100 g vs. 10.48 mg/100 g, respectively, *p* ≤ 0.05). Additionally, the hind leg showed higher concentrations of K, Na, and Mg compared to the loin.

Table 4 shows the percentage of dry matter, energy value, and protein, fat, and ash content in the meat of the two divergent lines selected by litter size variability, as well as in the hind legs, forelegs, and loin. The HO line had a lower dry matter percentage and a higher protein content compared to the HE line (30.25% vs. 33.12%, *p* ≤ 0.05, for dry matter; and 21.57 g/100 g vs. 20.75 g/100 g, *p* ≤ 0.05, for protein content). The forelegs had the highest energy value, fat content, and dry matter percentage compared to the hind legs and loin, while the hind legs and loin had the highest protein content compared to the forelegs (*p* ≤ 0.05). The ash content of the hind legs and loin was higher than that of the forelegs (*p* ≤ 0.05).

Table 5 shows the fatty acid profile in the meat of the two divergent lines selected by litter size variability and in the hind legs, forelegs, and loin. The fatty acid profile included saturated fatty acids (dodecanoic, tridecanoic, tetradecanoic, pentadecanoic, hexadecanoic, heptadecanoic, and octadecenoic), monounsaturated fatty acids (9-hexadecenoic acid, 6- and 10-octadecenoic acids, and 11-eicosenoic acid), and polyunsaturated fatty acids (9,12-octadecadienoic acid, 9,12,15-octadecatrienoic acid). Statistically significant differences were observed only in the total saturated fatty acid (SFA) content among the HO and HE lines and the carcass cuts. Rabbits from the HO line were characterized by 8% less SFA content than those from the HE line (*p* ≤ 0.05), while the hind legs and loin showed an 11% and 9% lower SFA content than that of the foreleg (*p* ≤ 0.05).

Table 6 shows the degree of fat oxidation in the meat of the two divergent lines selected by litter size variability and the hind legs, forelegs, and loin. Statistically significant differences in fat oxidation levels were observed between the different parts of the rabbit carcasses. The loin was characterized by the lowest degree of fat oxidation, followed by the hind legs, and the highest degree of fat oxidation was observed in the forelegs (*p* ≤ 0.05).

## 4. Discussion

### 4.1. Growth Performance and Carcass Traits

The determinations made in the carcass and meat quality were to assess the energy value, antioxidant properties, and fatty acid profile in the two divergent lines selected by litter size variability (the HO and HE lines). Comparing the HO and HE lines, which share the same genetic origin, helps analyze the potential impact of increasing litter size homogeneity on growth, carcass, and meat quality. We note that both lines have similar weights upon reaching adulthood [19]; therefore, comparison at the same age can be used as a good approximation, as suggested by Pascual et al. [18], and it has been employed in the studies of Piles et al. [29] and Hernández et al. [30].

Selection for homogeneity has been found to have a positive effect on litter size [16]. However, an increase in litter size leads to greater intrauterine competition among fetuses for maternal resources, resulting in reduced birth weight [14] and a negative impact on survival and subsequent postnatal development of kits [31]. Despite the HO line having larger litters than the HE line [16], we found a higher survival rate at birth and greater uniformity in weaning weight among kits from the HO line compared to those from the HE line in a previous study [32]. This was consistent with higher milk production and better maternal behavior during lactation in this line. Weight homogeneity within the litter is an important trait in prolific species such as rabbits, as increasing weight uniformity reduces competition between littermates and enhances their viability [17]. Additionally, in the present study, we observed that selection to increase homogeneity in litter size extended its positive effect after weaning, improving the animals’ growth rate at 63 days of age without negatively affecting carcass composition.

### 4.2. Meat Traits

Meat sensory and technological qualities are essential for consumer acceptability and have been shown to be closely linked to ultimate pH [33]. Consistent with the literature [29,34,35], our study found no significant effect of genotype on meat pH at 45 min and 24 h post-mortem. This suggests that selection for litter size homogeneity does not affect ultimate pH and, consequently, does not compromise the sensory or technological quality of rabbit meat. Regarding color lightness (L*), redness (a*), and yellowness (b*), the values obtained in our study were within the range reported in previous studies (54.8–61.1 for L*; 2.34–6.90 for a*; 1.05–8.10 for b*) [36,37,38,39,40,41]. Meat lightness (L*) has been associated with an increase in myofibrillar protein shrinkage, which is correlated with pH [42]. In this regard, meat from the HO line exhibited greater lightness than that from the HE line at slaughter; however, this difference disappeared at 24 h post-mortem due to a reduction in pH in both lines. Redness (a*) and yellowness (b*) of meat are influenced by myoglobin content, oxydo-reduction, the degree of iron atom oxidation, globin denaturation, and muscle fiber type [30,42]. In this study, we did not observe that selection for litter size homogeneity affected meat color, i.e., a* and b* values. In relation to the effect of muscle fiber type on pH and meat color, the hind legs had a higher pH value at 24 h post-mortem and displayed a darker color compared to the loin. A greater physical activity in the hind legs could contribute to higher myoglobin levels in this muscle, which may explain the higher pH and the darker meat color in this muscle type [36,42,43,44].

Regarding the mineral elements, the selection for litter size homogeneity did not modify K, Na, Mg, Zn, Fe, and Cu levels in rabbit meat but increased Ca levels compared to the selection for litter size heterogeneity. Few studies have examined the effect of muscle type on the concentrations of macro- and microminerals in rabbit meat. This study reported higher concentrations of K, Na, and Mg in the hind leg than in the loin. These minerals (K, Na, and Mg) are directly associated with greater contractile capacity, improved endurance, and reduced muscle fatigue. The higher concentrations of macro- and micro-minerals in the hind legs may be due to a higher level of physical activity in comparison to the loin [42,43,44]. These results are consistent with the lower energy levels we observed in the hind legs.

Healthy and dietetic meat is associated with high protein content, low levels of SFA (saturated fatty acids), and high levels of unsaturated fatty acids (MUFA and PUFA) [1]. In our study, we found that rabbit meat from the HO line exhibited a higher protein content and a lower SFA content compared to meat from the HE line. With respect to antioxidant properties, the selection process for homogeneity in litter size did not appear to have an adverse effect on the degree of fat oxidation. Consequently, selecting for homogeneity in litter size could potentially improve meat quality. Regarding the muscle type, the hind legs and loin had higher protein content, lower energy value, lower SFA content, and lower degree of fat oxidation compared to the forelegs. These results may be related to the fact that the hind legs and loin are primarily composed of fast-twitch muscle fibers, which are designed for faster movements [45,46,47]. These fibers store energy as glycogen rather than fat, leading to lower overall fat content, including SFA [48].

## 5. Conclusions

In this study, we found that selecting for increasing homogeneity in litter size does not compromise the growth rate, carcass composition, or quality of meat, such as pH and color. Besides, increasing litter size homogeneity had a positive effect on increasing protein content and decreasing SFA levels in rabbit meat. Furthermore, selection for homogeneity did not negatively affect the antioxidant properties of the meat. These findings suggest that selecting for litter size homogeneity can enhance meat quality, offering nutritional benefits without compromising productive performance. Selection for litter size homogeneity can be a key criterion in maternal lines, which are essential for obtaining hybrid commercial strains. Therefore, the results of this study may have favorable consequences for the development of new hybrid commercial strains.

## Figures and Tables

**Table 1:** Live weight, growth rate between weaning (30 days of age) and slaughter (63 days of age), and carcass traits in the line selected to increase (the HO line) and decrease (the HE line) litter size homogeneity.

	Line		
Trait	HO	HE	*p*-Value
Live weight at 30 d	787 ^a^	1124 ^b^	0.005
Live weight at 63 d	1777 ^a^	1965 ^b^	0.01
Increment in weight from 30 to 63 d	990 ^a^	841 ^b^	0.05
Growth rate from 30 to 63 d	30.9 ^a^	26.3 ^b^	0.05
Chilled carcass weight at 45 min p.m. (g)	1080	1166	ns
Chilled carcass weight at 24 h p.m. (g)	1056	1147	ns
Reference carcass weight (g)	877	943	ns
Dressing percentage (%)	59	58	ns
Meat part proportion (%)			
Liver percentage (%)	4.38	4.61	ns
Kidneys percentage (%)	1.15	1.10	ns
Fore legs percentage (%)	13.3	13.2	ns
Thoracic cage percentage (%)	12.0	11.1	ns
Loin percentage (%)	26.9	28.1	ns
Hind legs percentage (%)	29.2	28.5	ns
Fat proportion (%)			
Interscapular fat weight (%)	0.13	0.12	ns
Perirenal fat weigh (%)	0.49	0.61	ns
Inguinal fat weight (%)	0.04	0.07	ns

Different superscript letters (a and b) within row indicate significant differences at *p* ≤ 0.05. p.m.: post-mortem. ns: no significant difference.

**Table 2:** pH and color parameters at 45 min and 24 h post-mortem in the meat of the line selected to increase (the HO line) and decrease (the HE line) litter size homogeneity, as well as in the hind legs and loin.

	Line		Part		*p*-Value	
Trait	HO	HE	Hind Leg	Loin	Line	Part
pH^45 min p.m.^	6.72	6.65	6.45 ^a^	6.83 ^b^	ns	0.001
pH^24 h p.m.^	5.74	5.73	5.85 ^a^	5.66 ^b^	ns	0.001
L^*45 min p.m.^	58.8 ^a^	55.3 ^b^	55.1	54.3	0.01	ns
L^*24 h p.m.^	43.2	43.6	42.1 ^a^	44.4 ^b^	ns	0.001
a^*45 min p.m.^	5.17	4.57	6.25 ^a^	2.73 ^b^	ns	0.005
a^*24 h p.m.^	2.53	2.42	2.77 ^a^	2.21 ^b^	ns	0.05
b^*45 min p.m.^	8.93	8.97	10.51 ^a^	3.91 ^b^	ns	0.03
b^*24 h p.m.^	1.74	1.51	1.68	1.55	ns	ns

Different superscript letters (a and b) within row indicate significant differences at *p* ≤ 0.05. p.m.: post-mortem. ns: no significant difference.

**Table 3:** Levels of potassium (mg/100 g fresh meat), sodium (mg/100 g fresh meat), magnesium (mg/100 g fresh meat), calcium (mg/100 g fresh meat), zinc (mg/Kg fresh meat), iron (mg/Kg fresh meat), copper (mg/Kg fresh meat), and manganese (mg/Kg fresh meat) in the line selected to increase (the HO line) and decrease (the HE line) litter size homogeneity, as well as in the hind legs and loin.

	Line		Part		*p*-Value	
	HO	HE	Hind Leg	Loin	Line	Part
K	292.75	285.34	301.24 ^a^	281.05 ^b^	ns	0.04
Na	44.25	35.55	42.15 ^a^	33.74 ^b^	ns	0.04
Mg	30.39	28.01	31.51 ^a^	28.95 ^b^	ns	0.04
Ca	17.50 ^a^	10.48 ^b^	15.89	14.57	0.05	ns
Zn	11.63	8.08	11.12	9.54	ns	ns
Fe	10.44	7.66	10.92	6.98	ns	ns
Cu	1.09	0.42	0.77	0.66	ns	ns
Mn	0.22	0.12	0.19	0.16	ns	ns

Different superscript letters (a and b) within row indicate significant differences at *p* ≤ 0.05. ns: no significant difference.

**Table 4:** Dry weight (%), energy value (kcal/100 g), content in protein (g/100 g), fat (g/100 g), carbohydrate (g/100 g), and ash (g/100 g) in the meat of the line selected to increase (the HO line) and decrease (the HE line) litter size homogeneity, as well as in the hind legs, forelegs, and loin.

	Line		Part			*p*-Value	
Trait	HO	HE	Hind Leg	Foreleg	Loin	Line	Part
Dry weight (%)	30.25 ^a^	33.12 ^b^	29.70 ^a^	37.04 ^b^	30.92 ^a^	0.01	0.01
Energy value (kcal/100 g)	162.29	176.26	168.38 ^a^	194.70 ^b^	152.02 ^c^	ns	0.01
Protein content (g/100 g)	21.57 ^a^	20.75 ^b^	21.49 ^a^	19.11 ^b^	21.52 ^a^	0.01	0.01
Fat content (g/100 g)	2.52	2.27	1.56 ^a^	5.27 ^b^	2.52 ^c^	ns	0.01
Ash (g/100 g)	1.02	1.01	1.10 ^a^	0.94 ^b^	1.04 ^a^	ns	0.01

Different superscript letters (a, b and c) within row indicate significant differences at *p* ≤ 0.05. ns: no significant difference.

**Table 5:** Fatty acid profile in the meat of the line selected to increase (the HO line) and decrease (the HE line) litter size homogeneity, as well as in the hind legs, forelegs, and loin.

Trait	Line		Part			*p*-Value	
	HO	HE	Hind Leg	Foreleg	Loin	Line	Part
SFA	28.110 ^a^	36.220 ^b^	29.840 ^a^	40.820 ^b^	31.505 ^a^	0.01	0.01
MUFA	23.850	24.835	23.495	24.790	24.155	ns	ns
PUFA	25.680	26.425	25.795	26.300	25.905	ns	ns
n-3	1.890	1.825	1.685	2.010	1.915	ns	ns
n-6	22.780	24.535	22.145	23.290	24.260	ns	ns
n-6/n-3	12.050	13.220	13.663	11.810	12.052	ns	ns

Different superscript letters (a and b) within row indicate significant differences at *p* ≤ 0.05. ns: no significant difference.

**Table 6:** The degree of fat oxidation in the meat of the line selected to increase (the HO line) and decrease (the HE line) litter size homogeneity, as well as in the hind legs, forelegs, and loin.

Trait	Line		Part			*p*-Value	
	HO	HE	Hind Leg	Foreleg	Loin	Line	Part
TBA (mg malondialdehyde/kg of sample)	0.006	0.005	0.005 ^a^	0.025 ^b^	0.003 ^c^	ns	0.01

Different superscript letters (a, b and c) within row indicate significant differences at *p* ≤ 0.05. ns: no significant difference.

## Data Availability

The data presented in this study are available on request from the corresponding author due to privacy.
